# AKR1C3 in carcinomas: from multifaceted roles to therapeutic strategies

**DOI:** 10.3389/fphar.2024.1378292

**Published:** 2024-03-08

**Authors:** Mengnan Li, Limin Zhang, Jiahui Yu, Xiaoxiao Wang, Le Cheng, Zhaowu Ma, Xiaoguang Chen, Lingzhi Wang, Boon Cher Goh

**Affiliations:** ^1^ School of Basic Medicine, Health Science Center, Yangtze University, Jingzhou, China; ^2^ Jingzhou Hospital of Traditional Chinese Medicine, Jingzhou, China; ^3^ The Third Clinical Medical College of Yangtze University, Jingzhou, China; ^4^ Department of Haematology–Oncology, National University Cancer Institute, Singapore, Singapore; ^5^ NUS Center for Cancer Research (N2CR), Yong Loo Lin School of Medicine, National University of Singapore, Singapore, Singapore; ^6^ Department of Pharmacology, Yong Loo Lin School of Medicine, National University of Singapore, Singapore, Singapore; ^7^ Cancer Science Institute of Singapore, National University of Singapore, Singapore, Singapore; ^8^ Department of Medicine, Yong Loo Lin School of Medicine, National University of Singapore, Singapore, Singapore

**Keywords:** AKR1C3, carcinoma progression, therapeutic resistance, inhibitors, combination therapies

## Abstract

Aldo-Keto Reductase Family 1 Member C3 (AKR1C3), also known as type 5 17β-hydroxysteroid dehydrogenase (17β-HSD5) or prostaglandin F (PGF) synthase, functions as a pivotal enzyme in androgen biosynthesis. It catalyzes the conversion of weak androgens, estrone (a weak estrogen), and PGD2 into potent androgens (testosterone and 5α-dihydrotestosterone), 17β-estradiol (a potent estrogen), and 11β-PGF2α, respectively. Elevated levels of AKR1C3 activate androgen receptor (AR) signaling pathway, contributing to tumor recurrence and imparting resistance to cancer therapies. The overexpression of AKR1C3 serves as an oncogenic factor, promoting carcinoma cell proliferation, invasion, and metastasis, and is correlated with unfavorable prognosis and overall survival in carcinoma patients. Inhibiting AKR1C3 has demonstrated potent efficacy in suppressing tumor progression and overcoming treatment resistance. As a result, the development and design of AKR1C3 inhibitors have garnered increasing interest among researchers, with significant progress witnessed in recent years. Novel AKR1C3 inhibitors, including natural products and analogues of existing drugs designed based on their structures and frameworks, continue to be discovered and developed in laboratories worldwide. The AKR1C3 enzyme has emerged as a key player in carcinoma progression and therapeutic resistance, posing challenges in cancer treatment. This review aims to provide a comprehensive analysis of AKR1C3’s role in carcinoma development, its implications in therapeutic resistance, and recent advancements in the development of AKR1C3 inhibitors for tumor therapies.

## 1 Introduction

AKR1C3 has recently emerged as a key contributor to the accelerated proliferation and metastasis observed in carcinomas. Within the aldo-keto reductase (AKR) superfamily, AKR1C3 functions as a hormone activity regulator and PGF synthase, exerting influence over hormone receptor occupancy and cellular proliferation (Liu et al., 2020). Distinguished as 17β-HSD5, AKR1C3 is notable as the sole human 17β-HSD not classified as a short-chain dehydrogenase/reductase (Penning, 2019).

The AKR1C subfamily encompasses four isoforms (AKR1C1-AKR1C4), with AKR1C3 sharing substantial sequence homology (>86%) with AKR1C1, AKR1C2, and AKR1C4 (Liu et al., 2020). Despite their structural similarities, these isoforms exhibit distinct distribution preferences and biological functions. AKR1C3, with varying ratios of 3-keto, 17-keto, and 20-ketosteroid reductase activities, prominently functions as a 17-ketosteroid reductase, demonstrating the highest catalytic efficiency in converting delta4-androstenedione to testosterone (Byrns et al., 2010; Penning, 2019).

Overexpression of AKR1C3 is evident at both mRNA and protein levels across diverse carcinoma types, encompassing hormone-dependent and hormone-independent cancers. Elevated AKR1C3 levels correlate with increased cancer cell growth, proliferation, migration, and metastasis. Inhibiting AKR1C3 through specific inhibitors has proven effective in halting carcinoma progression, restoring sensitivity to cancer therapies, and enhancing overall prognosis.

The heightened focus on AKR1C3 as a therapeutic target has garnered global interest, driving ongoing investigations into various AKR1C3 inhibitors across laboratories worldwide. These inhibitors, including steroids, non-steroidal anti-inflammatory drugs, and diverse natural products, exhibit potential therapeutic efficacy. Notably, Indomethacin, a widely used non-steroidal anti-inflammatory drug, stands out for its potent AKR1C3 inhibitory properties. This review aims to provide a comprehensive analysis of AKR1C3’s role in carcinoma development, its implications in therapeutic resistance, and recent advancements in the development of AKR1C3 inhibitors for tumor therapies. Different from mainstream studies focusing on the function of AKR1C3 in hormone-dependent cancers, this review pays more attention to hormone-independent cancers and the combined use of AKR1C3 inhibitors and conventional treatment regimens in antineoplastic protocols. More importantly, we concentrate on systematic summary of AKR1C3 inhibitors and AKR1C3-related clinical trials.

## 2 Literature search and screening

**FIGURE 1:**
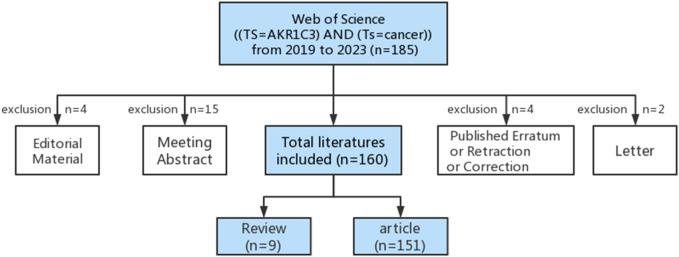
Schematic diagram of literature retrieval.

We obtained literature resources by querying the Web of Science database using the search condition “AKR1C3 (Topic) AND cancer (Topic)” within the time frame from 1 January 2019 to 25 March 2023. A total of 185 results were initially retrieved. To focus on the review theme, we refined the selection by excluding certain document types such as Editorial Material and Letter. After this refinement, 160 documents closely related to the review theme were retained. The specific search process is illustrated in Figure 1.

## 3 AKR1C3 in carcinoma progression of hormone-dependent cancers

**FIGURE 2:**
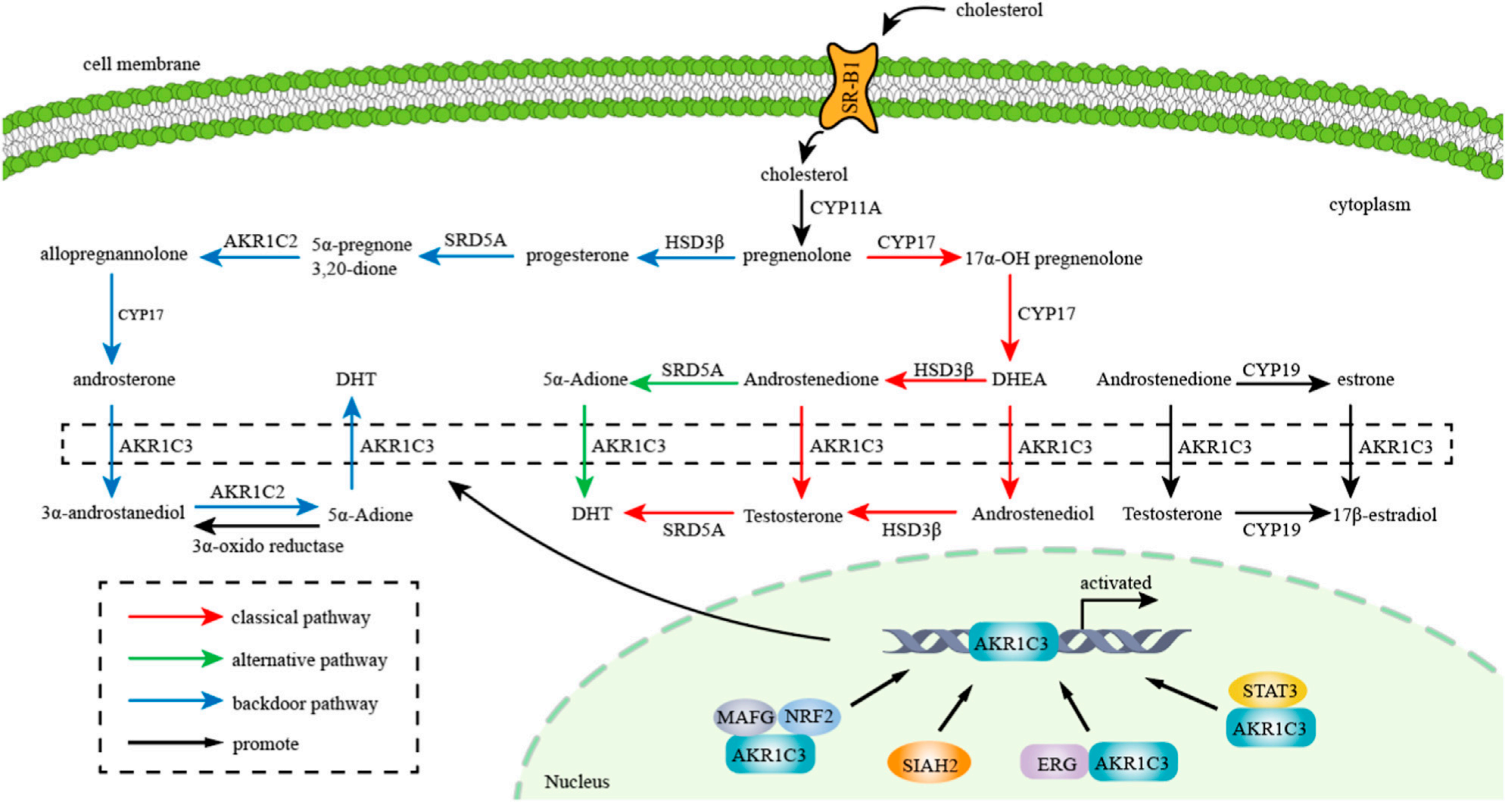
Metabolism of steroid hormones mediated by AKR1C3: AKR1C3 catalyzes weak androgen and estrogen to potent androgen (Testosterone and DHT) and estrogen (17β-estradiol) respectively. “red arrow” represents classical pathway of androgen metabolism, “green arrow” represents alternative pathway of androgen metabolism, and “blue arrow” represents backdoor pathway of androgen metabolism. Transcription factors such as NRF2, SIAH2, ERG and STAT3 promote AKR1C3 expression and thereby facilitate metabolism of gonadal hormones.

**FIGURE 3:**
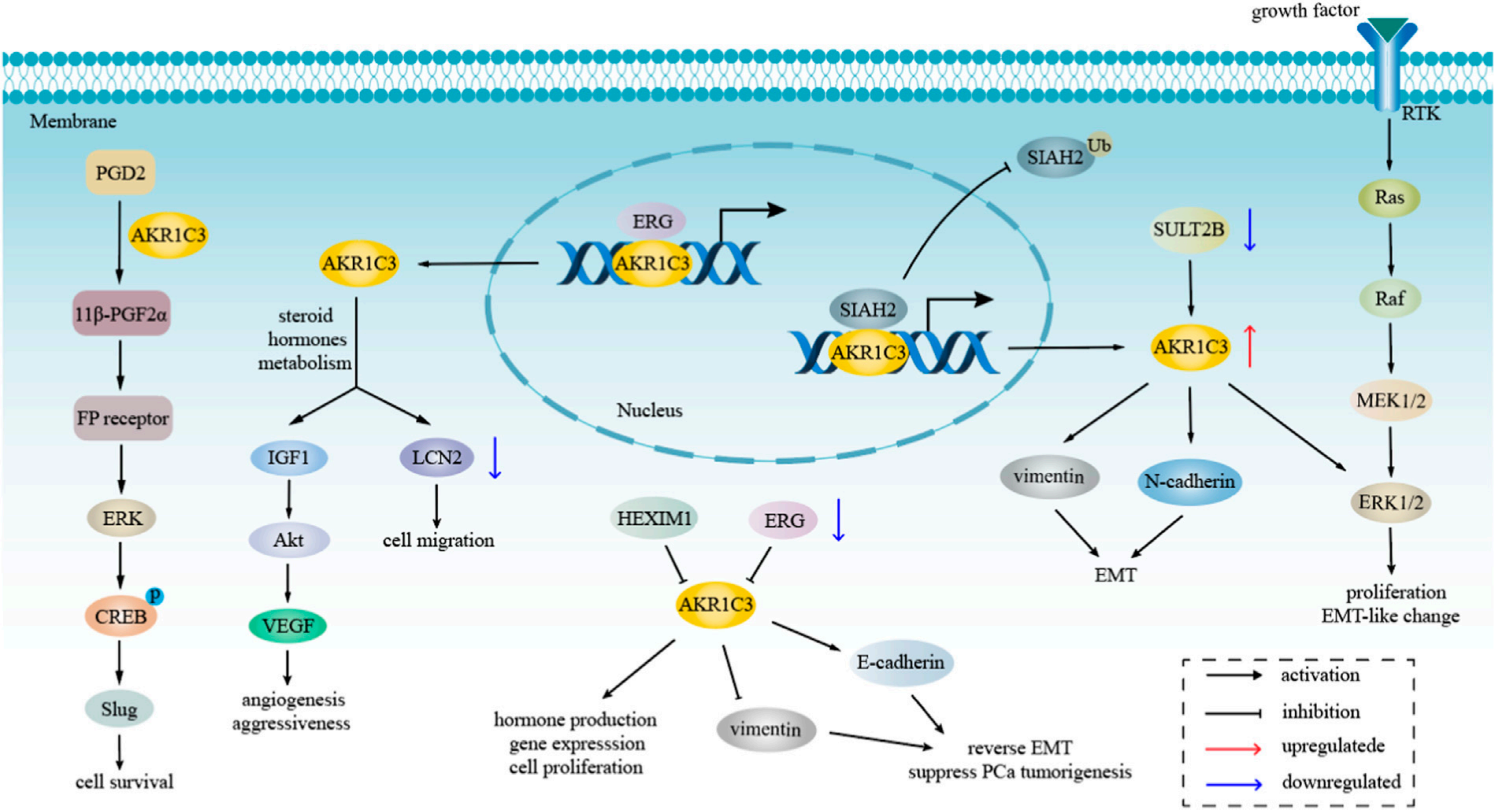
The role of AKR1C3 in hormone-dependent tumors progression. AKR1C3 regulates LCN2 and Akt pathway through involving in steroid hormones metabolism, resulting in cell migration, tumor angiogenesis and aggressiveness. AKR1C3 not only mediates cell survival and proliferation via ERK pathway, but also modulates EMT by regulating the level of vimentin, E-cadherin, and N-cadherin.

AKR1C3 exhibits expression in various endocrine organs, including the prostate, adrenals, breast, and uterus, suggesting its involvement in hormone-dependent and hormone-independent cancer growth by influencing both steroid hormone and prostaglandin-mediated signaling pathways (Byrns et al., 2008; Yepuru et al., 2013). The metabolism of steroid hormones mediated by AKR1C3 and its transcription factors are demonstrated in Figure 2. Additionally, the potential role of AKR1C3 in the progression of hormone-dependent tumors was depicted in Figure 3. This section explores the role of AKR1C3 in specific types of hormone-dependent cancers:

### 3.1 Prostate cancer

Prostate cancer (PCa) is a prevalent malignancy within the male genital system, primarily afflicting elderly men, characterized as an androgen-dependent tumor. Its onset and progression hinge upon androgen levels. The AKR1C3 gene assumes a pivotal role in androgen formation and metabolism, specifically catalyzing the synthesis of testosterone and dihydrotestosterone (DHT) within tumor cells (Yu et al., 2023). In a study by Cui et al., an analysis of 496 PCa patients from The Cancer Genome Atlas (TCGA) database revealed a correlation between increased AKR1C3 expression and advanced disease stages, as indicated by high T stage, N stage, and Gleason score values. However, age did not exhibit a similar correlation, suggesting that AKR1C3 may serve as a prognostic indicator for poor outcomes among PCa patients (Cui et al., 2023). Notably, AKR1C3 levels were comparable in primary PCa and normal prostate, but significantly elevated in metastatic PCa based on data from GEO datasets and Oncomine (Liu et al., 2015). Moreover, AKR1C3 was found to be associated with metastatic status in human PCa cells, suggesting its potential role in cancer cell proliferation, migration, and resistance to enzalutamide (Neuwirt et al., 2020).

The cornerstone of treating advanced PCa is androgen-deprivation therapy (ADT), aiming to reduce circulating androgens to castration levels (Polotti et al., 2017). Despite its efficacy, a substantial number of hormone-sensitive PCa cases eventually develop resistance to ADT, progressing into castration-resistant PCa (CRPC), often within the first year of ADT in men with metastatic PCa (Varenhorst et al., 2016). CRPC, a fatal and metastatic manifestation, may be influenced by the overexpression of AKR1C3. Consequently, inhibiting AKR1C3 emerges as a promising strategy for CRPC treatment. The primary mechanism of CRPC progression involves cancer cells utilizing androgenic precursors from the blood or synthesizing *de novo* testosterone through the upregulation of steroidogenic enzymes, including Cyp17A1, AKR1C3, and 5α-reductase.

Powell et al. demonstrated that ERG regulates the expression of AKR1C3 in PCa cells by direct binding to the AKR1C3 gene. Knockdown of ERG resulted in reduced AKR1C3 expression, leading to decreased DHT synthesis and prostate-specific antigen (PSA) expression in VCaP PCa cells treated with 5α-androstanedione (5α-Adione). The positive correlation between the level of AKR1C3 expression and an elevated Gleason score suggests its potential as a biomarker for PCa progression (Powell et al., 2015). Additionally, AKR1C3 was identified as one of the most downregulated genes in the steroid hormone biosynthesis signaling pathway, possibly contributing to the transition from androgen dependence to CRPC. The ADAM9/UBN2/AKR1C3 axis, supported by the WCM dataset, was proposed as a novel regulatory mechanism in the transition of androgen-dependent PCa cells to an androgen-independent state during ADT. This axis, involving ADAM9 and UBN2, may represent a therapeutic target for improving ADT (Le et al., 2022).

AKR1C3 influences PCa cell growth and proliferation through diverse mechanisms and pathways. It catalyzes PGD2 into 11β-PGF2α, generating proliferative signals that promote prostate cell growth. Studies by Wang and colleagues suggest that both AKR1C2 and AKR1C3 mediate similar PGD2 conversion, promoting prostate cell proliferation through FP and PI3K/Akt signaling pathways (Wang et al., 2008). Moreover, AKR1C3 was identified as an AR-selective coactivator, interacting with AR in PCa cells, xenografts, and human CRPC samples. This interaction facilitated the growth of both androgen-dependent PCa and CRPC xenografts, concomitant with the reactivation of androgen signaling (Yepuru et al., 2013). The complex formed by AKR1C3 and AR-V7 was found to be crucial for tumor growth in CRPC cells after ADT treatment, suppressing the protein degradation of both components (Wang et al., 2020a). Targeting AKR1C3 using aptamer-PSMA guided siAKR1C3@PPA that is assembled from PEG3500, PAMAM, and siRNA for AKR1C3 into PSMA-positive PCa cells, resulting in specific downregulation of AKR1C3 and potential cell cycle arrest (Cui et al., 2022). Additionally, HEXIM1 was identified as a regulator, downregulating AKR1C3 expression in breast and PCa cells, affecting hormone production, gene expression, and cell proliferation (Mozar et al., 2022).

Observations by Fan et al. highlighted parallel expression of Siah2 and AKR1C3 in human PCa tissues, correlating with increasing Gleason grade and poor prognosis. AKR1C3 functions as a downstream effector of Siah2, driving PCa growth *in vitro* and *in vivo* independently of its catalytic activity. Siah2 inhibition decreased AKR1C3 expression, intracellular androgen levels, and inhibited cell growth, while AKR1C3 re-expression in Siah2 knockdown cells elevated Siah2 protein levels. Surprisingly, AKR1C3’s activities did not inhibit Siah2’s degrading function of other targets (Fan et al., 2015). Moreover, AKR1C3 expression was significantly associated with EMT in human PCa specimens from public tissue microarrays. Inhibition of AKR1C3 was found to upregulate E-cadherin expression, downregulate vimentin, and suppress PCa tumorigenesis *in vitro* and reverse EMT *in vivo* (Wang et al., 2018). Another study demonstrated that AKR1C3 overexpression increased the expression of N-cadherin and vimentin while decreasing E-cadherin expression, further supporting its role in EMT (Cui et al., 2023). In CRPC cells, depletion of SULT2B, a prostate-expressed hydroxysteroid sulfotransferase, upregulated AKR1C3, activated ERK1/2 survival signal, and induced EMT-like changes (Park et al., 2020). This information suggests that pathways regulating the inhibitory SULT2B-AKR1C3 axis may present new avenues for targeting SULT2B-deficient PCa.

AKR1C3 promoted EMT by regulating transcription factors and signaling pathways and led tumor cells to acquire more aggressive and metastatic characteristics of mesenchymal cells (Thiery et al., 2009). Beyond its involvement in cell proliferation and EMT, AKR1C3 has been implicated in angiogenesis. AKR1C3 has been demonstrated to facilitate PCa cells angiogenesis and aggressiveness through upregulating the expression level of insulin-like growth factor (IGF)-1, Akt, and vascular endothelial growth factor (VEGF) from bioinformatics analysis and functional genomics (Dozmorov et al., 2010).

### 3.2 Breast cancer

AKR1C3 appears to play crucial roles in the development of hormone-dependent breast cancer, and potentially hormone-independent breast cancer. It is involved in the reduction of prostaglandins, which may generate hormone-independent proliferative signals (Byrns and Penning, 2009). The combined impact of AKR1C3-catalyzed 17- and 20-ketosteroid reductions could elevate the 17β-estradiol to progesterone ratio in the breast, potentially increasing estrogen receptor (ER)α and decreasing progesterone receptor (PR) signaling, although this hypothesis requires further investigation. Furthermore, the formation of PGF2 epimers by AKR1C3 could activate F prostanoid receptors and compromise PPARγ's putative anti-proliferative PGJ2 ligands. Hence, AKR1C3 emerges as a source of proliferative signals and a potential therapeutic target for both hormone-dependent and hormone-independent breast cancer (Byrns and Penning, 2009). The increased PPARγ-dependent and decreased NFκB-dependent and ERα-dependent gene transcription resulting from AKR1C3 inhibition is predicted to inhibit breast cancer cell proliferation, making it a potential target for breast cancer treatment.

Given its potential contribution to breast cancer cell proliferation, AKR1C3 represents a promising therapeutic target. In immunohistochemical analysis, FP receptor status was associated with adverse clinical outcomes only in cases positive for AKR1C3. Treatment with 11β-PGF2α phosphorylated ERK and CREB, inducing Slug expression through FP receptor activation in MCF-FP cells. These cells exhibited decreased chemosensitivity compared to parental controls (Yoda et al., 2015). These findings suggest that AKR1C3 actions can produce FP receptor ligands, activating pathways that promote carcinoma cell survival in breast cancer.

A meta-analysis of AKR1C3 mRNA expression in patient samples revealed upregulation in CRPC but downregulation in ER-positive breast cancer (Yin et al., 2014). Lewis et al. reported downregulated expression of AKR1C3 in breast cancer compared to normal breast tissue (Lewis et al., 2004). Additionally, a negative relationship was observed between the expression of 17β-HSD5 and the apoptosis inhibitor GRP78. Knockdown or inhibition of 17β-HSD5 increased cell viability and proliferation (Xu et al., 2017). Despite inconsistent reports on 17β-HSD5 expression and its prognostic value in breast cancer, it may not be a latent therapeutic target, but its low expression could serve as a poor prognosis factor.

Triple-negative breast cancer (TNBC), characterized by negative ER, PR, and human epidermal growth factor receptor-2 (HER-2), presents unique biological behaviors with poor prognosis due to the lack of effective targeted therapy. Chemotherapy is comparatively more effective for TNBC than other subtypes. McNamara et al. reported a notable positive association between AR and 17β-HSD5 in TNBC, verified in a public microarray dataset (McNamara et al., 2013; McNamara et al., 2014). AR deletion in TNBC was linked to a more aggressive phenotype, suggesting that downregulation of AR and androgen-metabolizing enzymes might contribute to increased biological aggressiveness in TNBC development.

### 3.3 Gynecological malignant tumors

In various human cancers, AKR1C3 has exhibited upregulation, with particular significance in endometrial cancer, the most prevalent malignancy of the female genital tract. Evidence suggests that the expression of enzymes, including aromatase, sulphatase, and AKR1C3 by endometrial cells, plays a pivotal role in tissue function and malfunction (Gibson et al., 2020). Tea et al. proposed that AKR1C3 expression may elevate estradiol concentration within the endometrium, contributing to enhanced estrogen action (Rizner et al., 2006). Notably, AKR1C3 expression was observed to be higher in endometrial cancer compared to ovarian cancer. In endometrioid endometrial carcinoma, elevated AKR1C3 expression, as indicated by immunohistochemistry (IHC), was correlated with improved overall survival and disease-free survival. This suggests that AKR1C3 holds promise as a potential prognostic biomarker for endometrioid endometrial cancer (Hojnik et al., 2020).

Furthermore, in uterine cervical cancer, AKR1C3 overexpression was associated with reduced LCN2 promoter activity and LCN2 expression, leading to enhanced cell migration. Conversely, AKR1C3 silencing upregulated LCN2 expression, resulting in decreased cell migration, invasion, and cytoskeleton changes in cervical cancer cells. The positive correlation between AKR1C3 and negative LCN2 expression was linked to higher recurrence and poorer survival in cervical cancer patients (Wu et al., 2014). These findings underscore the complex role of AKR1C3 in gynecological malignancies and its potential as a prognostic indicator in specific subtypes of endometrial and cervical cancers.

## 4 AKR1C3 in carcinoma progression of hormone-independent cancers

**FIGURE 4:**
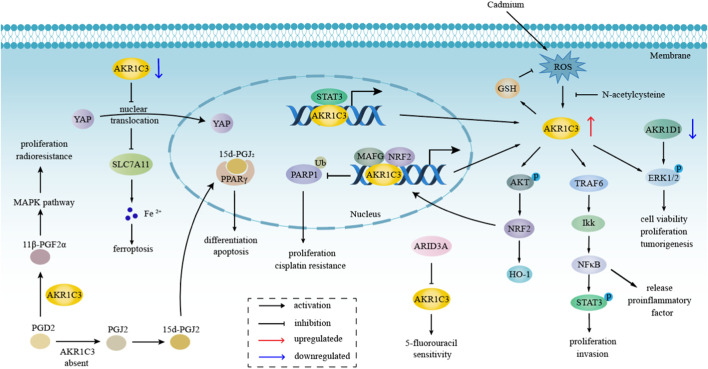
The role of AKR1C3 in hormone-independent tumors progression. AKR1C3 takes part in the process of hormone-independent cancers, including proliferation, invasion, tumorigenesis, etc., principally through NFκB, MAPK, ERK, and Akt pathway, which are also related to therapeutic resistance. Downregulation of AKR1C3 promotes ferroptosis through the YAP/SLC7A11 pathway. The metabolism of PGD2 depends on whether AKR1C3 was present or not. When AKR1C3 is present, PGD2 is catalyzed to 11β-PGF2α, which influenced MAPK pathway.

Over the preceding decade, the pivotal involvement of AKR1C3 in the advancement of hormone-independent cancers has garnered substantial attention within the realm of cancer research. Figure 4 elucidates the potential role of AKR1C3 in the progression of hormone-independent tumors.

### 4.1 Liver cancer

Primary liver cancer, encompassing hepatocellular carcinoma (HCC) and cholangiocellular carcinoma, poses a formidable clinical challenge due to its poor prognosis, characterized by a high metastasis rate and recurrence frequency. Investigation into the role of AKR1C3 in the context of liver cancer progression has provided valuable insights.

AKR1C3 exhibits significantly elevated expression levels in both liver cancer tissues and cells, as discerned through analyses utilizing the Oncomine, CCLE, and GEPIA databases (Zhao et al., 2019). Bioinformatics methodologies indicate an overexpression of AKR1C3, accompanied by a downregulation of AKR1D1, correlating with unfavorable prognosis and a shortened median survival time in HCC.

AKR1C3 and AKR1D1 are implicated in the MAPK/ERK and AR signaling pathways. Experimental manipulations, such as AKR1C3 knockdown or AKR1D1 overexpression, result in reduced levels of AR and phosphorylated ERK1/2, leading to inhibited cell viability, proliferation, and tumorigenesis (Zhu et al., 2021). This suggests a potential oncogenic role for AKR1C3 in HCC progression through modulation of the MEK/ERK and AR signaling pathways. Moreover, AKR1C3 regulates and activates NF-κB by inducing autoubiquitination of TRAF6, subsequently releasing proinflammatory factors that enhance STAT3 phosphorylation, fostering increased tumor cell proliferation and invasion. Additionally, AKR1C3 promotes tumor proliferation and invasion through the IL6/STAT3 pathway, as evidenced by gain- and loss-of-function experiments. A positive regulatory feedback loop is established through direct binding of STAT3 to the AKR1C3 promoter (Zhou et al., 2021b).

The NRF2/MAFG-AKR1C3-PARP1 axis also emerges as a vital pathway associated with proliferation in HCC. NRF2/MAFG directly binds to the AKR1C3 promoter, activating transcription. AKR1C3 stabilizes PARP1 by decreasing its ubiquitination, contributing to HCC cell proliferation and low sensitivity to Cisplatin (Pan et al., 2022). AKR1C3 contributes to the accumulation of lipid droplets (LD) in HCC, a phenomenon associated with cancer metastasis, stem cell proliferation, and chemoresistance across various tumor types (Wu et al., 2022). In addition, AKR1C3 regulates ferroptosis through the YAP/SLC7A11 signaling pathway in HCC. Downregulation of AKR1C3 leads to decreased YAP nuclear translocation, inhibiting the cystine transporter SLC7A11, thereby increasing intracellular ferrous iron levels and promoting ferroptosis (Chen et al., 2023). Furthermore, the combination of AKR1C3 and SLC7A11 is identified as a potent predictor of poor prognosis in HCC, highlighting the clinical relevance of these molecular interactions (Chen et al., 2023). This comprehensive analysis elucidates the intricate role of AKR1C3 in the progression of hormone-independent liver cancer, shedding light on potential avenues for therapeutic intervention and prognostic assessment.

### 4.2 Acute myeloid leukemia

Acute leukemia (AL) encompasses two primary subtypes: acute lymphoblastic leukemia (ALL) and acute myeloid leukemia (AML). AML is a hematological malignancy arising from hematopoietic stem progenitor cells, characterized by abnormal proliferation of primitive myeloid cells in bone marrow and peripheral blood (Garg et al., 2015; Short et al., 2018). The expression of AKR1C3 in AML has been associated with the efficacy of standard induction therapies, particularly in cases where AKR1C3 metabolizes drugs such as anthracyclines (Morell et al., 2020).

Despite AKR1C3 not being overexpressed in leukemia compared to non-leukemic or healthy tissues, an increase in AKR1C3 expression has been observed in T-cell acute lymphoblastic leukemia (T-ALL) compared with other leukemia subtypes, including AML and B-cell acute lymphoblastic leukemia (B-ALL) (Morell et al., 2020). Leukemia stem cell (LSC) markers, including AKR1C3, CD34, and MMRN1, were found to be upregulated in the high T-cell immunoglobulin mucin-3 (Tim3) group of TCGA. Tim-3 expression in LSCs has been linked to poor prognosis and distinctive biological features in AML (Wu et al., 2023).

AKR1C3, acting as a PGF2 synthase in leukemia, promotes cellular proliferation and impedes myeloid differentiation. Under AKR1C3 influence, PGD2 is catalyzed into 11β-PGF2α, blocking myeloid differentiation and facilitating cell proliferation through the MAPK signaling pathway. In the absence of AKR1C3, PGD2 converts to PGJ2 or 15-deoxy-Δ-PGJ2, assumed ligands of PPARγ, promoting differentiation and apoptosis through PPARγ activation (Verma et al., 2016; Penning, 2019; Penning et al., 2021).

Preclinical studies involving specific AKR1C3 prodrugs, such as AST-006 (TH-3424), have demonstrated cytotoxicity and anti-tumor activities against T-ALL cells *in vitro* and *in vivo* with AKR1C3 overexpression (Wang et al., 2020b; He et al., 2021). Additionally, prodrugs like PR104A and OBI-3424, activated by AKR1C3, exhibit efficacy in preclinical models of T-ALL. The clinical potential of OBI-3424 is currently being evaluated in phase 1/2 trials for patients with HCC or CRPC (ClinicalTrials.gov NCT03592264) (Evans et al., 2019; Reddi et al., 2022). Furthermore, PR-104 has been shown to reduce all cells with high AKR1C3 expression in the bone marrow rapidly, making it a compelling treatment strategy for T-ALL patients characterized by AKR1C3 overexpression and hypoxia (Moradi Manesh et al., 2015). AKR1C3 serves as a biomarker indicating the sensitivity of T-ALL to PR-104/PR-104A in both *in vivo* and *in vitro* settings.

### 4.3 Gastrointestinal cancer

Gastrointestinal cancer represents a prevalent and significant category of malignant tumors, encompassing gastric cancer, colon cancer, and rectal cancer. Steroid hormones play a crucial role in gastric carcinogenesis, with substantial local production in the peripheral tissues of both genders. In a study led by Frycz and colleagues, the measurement of AKR1C3 transcript and protein levels in non-tumoral and primary tumoral gastric tissues revealed an association with clinicopathological features of gastric cancer (GC). The histone deacetylase inhibitor sodium butyrate (NaBu) was found to elevate AKR1C3 transcript and protein levels in GC cell lines (EPG 85-257 and HGC-27), suggesting a potential involvement of decreased AKR1C3 expression in GC development, restorable by NaBu (Frycz et al., 2016). Additionally, Matsunaga et al. reported doxorubicin-reductase activity exhibited by AKR1C3 in gastrointestinal cancer cells (Matsunaga et al., 2019).

While AKR1C3 expression is generally upregulated in various cancer types, it has been found to be downregulated in colon cancer tissues compared to normal tissues. Li Yafei and colleagues identified ARID3A as a transcription factor for AKR1C3, inhibiting its expression in colon cancer cells. This interaction between AKR1C3 and ARID3A was associated with decreased chemosensitivity to 5-fluorouracil (5-FU) in colon cancer cells, suggesting that the ARID3A to AKR1C3 ratio may serve as a valuable marker for predicting the prognosis of colon cancer patients (Li et al., 2022).

Esophageal adenocarcinoma (EAC) and squamous cell carcinoma (ESCC) stand as the two primary histological subtypes of esophageal cancer. In multiple public datasets, the expression of AKR1C3 is notably heightened in EAC compared to normal esophageal tissue. Significantly, AKR1C3 has been implicated in promoting proliferation, colony formation, and migration in EAC cell lines, underscoring its pivotal role in EAC development (Zhou et al., 2021a). The positive regulation of AKT phosphorylation by AKR1C3, a key component in multiple signaling pathways, further emphasizes its involvement in diverse cellular processes (Manning and Toker, 2017). Additionally, AKR1C3 positively regulates glutathione (GSH), reinforcing cellular antioxidant defense and contributing substantially to protection against chemotherapy-induced toxicity. In essence, AKR1C3 appears to mitigate intracellular reactive oxygen species (ROS) levels in EAC cells through the AKT/GSH signaling axis. Notably, AKR1C3 is identified as a direct target of NRF2, a critical regulator of redox balance (Zhou et al., 2021a).

### 4.4 HPV-negative oropharynx squamous cell carcinoma

AKR1C3 demonstrates its highest transcriptional levels in HPV-negative oropharynx squamous cell carcinoma (OPSCC) samples, as indicated by data from study (Peraldo-Neia et al., 2021). Notably, the expression of AKR1C3 correlates positively with poorer survival outcomes in a cohort of 111 independent OPSCC cases, encompassing both the entire cohort and HPV-positive samples. The inhibition of AKR1C3 has shown the potential to enhance the effectiveness of Cisplatin, positioning AKR1C3 as a promising prognostic biomarker and a viable drug target in OPSCC.

### 4.5 Thyroid cancer

Apart from its involvement in colon cancer, the knockdown of AKR1C3 has been substantiated to promote proliferation, invasion, and migration abilities in thyroid cancer cell lines (Wang et al., 2022). This suggests that AKR1C3 may play a role as a progression promoter in thyroid cancer.

### 4.6 Lung cancer

The expression of AKR1C3 in human tumor surgical samples exhibits heterogeneity across various cancer types. In the context of lung cancer, the uniqueness lies in the exclusive expression of AKR1C3 in non-small cell lung cancer (NSCLC), with a negative expression observed in small cell lung cancer (Guise et al., 2010).

### 4.7 Nasal septum carcinoma

Cadmium, a toxic metal, induces AKR1C3 expression in the context of human nasal septum carcinoma. The utilization of N-acetyl cysteine (NAC) and the PI3K inhibitor (Ly294002) successfully inhibits the upregulation of AKR1C3 protein induced by cadmium. The evidence from Western blot and mRNA-differential display suggests that cadmium induces AKR1C3 expression at the transcription/translation stage, with this process requiring the mediation of the PI3K/Akt pathway (Lee et al., 2011).

## 5 Therapeutic resistance and AKR1C3

AKR1C3, overexpressed in various cancer tissues, has been associated with disease progression and poor prognosis, playing a pivotal role in resistance to multiple cancer treatment modalities, including radiation, chemotherapy drugs, and steroid drugs.

### 5.1 Radiotherapy resistance

In the context of radiotherapy resistance, AKR1C3 exhibits elevated expression in radioresistance-acquired cells, a phenomenon rarely observed in most human tissues. The heightened expression of AKR1C3 serves to clear ROS and promotes the accumulation of PGF2α. This dual action not only fosters the proliferation of PCa cells but also contributes to the radioresistance of these cells by activating MAPK signaling pathway (Xiao et al., 2021). Importantly, AKR1C3 demonstrates efficacy comparable to NAC, a ROS scavenger, in alleviating oxidative stress and reducing DNA damage caused by ionizing radiation, ultimately leading to radioresistance (Xiong et al., 2014). Chemical inhibition of AKR1C3, such as with the indomethacin inhibitor, has been shown to restore sensitivity to radiation in acquired tumor cells, highlighting AKR1C3 as a potential target for overcoming radioresistance (Sun et al., 2016). Overall, the overexpression of AKR1C3 significantly enhances resistance to radiation in human PCa cells through the activation of the MAPK pathway.

Moreover, in NSCLC, AKR1C3-mediated radioresistance may be linked to a decrease in G2/M phase arrest associated with radiation therapy and radiation-induced apoptosis (Xie et al., 2013). High nuclear expression of AKR1C3 and β-catenin in NSCLC has been correlated with radiation resistance, where increased nuclear expression of AKR1C3 is associated with worse short-term curative effects after radiotherapy. The nuclear aggregation of AKR1C3 during NSCLC radiation resistance suggests a potential synergistic relationship with the nuclear aggregation of β-catenin (Xiong and Hu, 2021). These findings underscore the intricate involvement of AKR1C3 in radiotherapy resistance across different cancer types, emphasizing its significance as a potential therapeutic target.

### 5.2 Chemotherapy resistance

Chemotherapy, a systemic treatment approach targeting various organs and tissues, is categorized into alkylating agents, antimetabolites, antibiotics, botanicals, hormones, and miscellaneous agents. Despite its effectiveness, chemotherapy resistance remains a significant challenge, and AKR1C3 has emerged as a key player in mediating resistance to chemotherapeutic drugs.

The TCGA dataset reveals that drug-metabolism-related enzymes are upregulated in the AKR1C3-high group, suggesting a potential role of AKR1C3 in chemotherapy resistance (Zhou et al., 2021a). In Signet Ring Cell Gastric Carcinoma (SRCGC), elevated levels of AKR1C1 and AKR1C3 reduce cisplatin-induced cell death by regulating intracellular ROS, thereby contributing to cisplatin resistance (Phoo et al., 2021). Combining inhibitors of AKR1C3, glutathione synthesis, and/or proteasomal proteolysis has proven effective in enhancing cisplatin sensitivity in breast cancer cells (Kobayashi et al., 2022).

In colon cancer, ARID3A, a transcriptional regulator, inhibits AKR1C3 transcription, leading to downregulation of AKR1C3. This downregulation enhances chemosensitivity to 5-FU, an antimetabolite, and patients with a higher ARID3A to AKR1C3 ratio exhibit a better prognosis (Li et al., 2022). Additionally, AKR1C3 mediates the inactivation and resistance of anthracycline drugs, such as doxorubicin, through its carbonyl reductase activity (Liu et al., 2020). The reduced form of doxorubicin, resulting from AKR1C3 activity, exhibits lower affinity and capacity to bind to DNA (Heibein et al., 2012).

In EAC, AKR1C3 regulates cellular ROS levels via the AKT signaling pathway, contributing to chemotherapy resistance. Targeting AKR1C3 is proposed as a novel strategy to sensitize EAC cells to conventional chemotherapy and improve patient survival (Sies and Jones, 2020; Zhou et al., 2021a). Furthermore, AKR1C3 is implicated in chemotherapy resistance to paclitaxel (PTX), a botanical agent, by promoting the metabolism of cytotoxic aldehyde. Combining AKR1C3 inhibitors with ATP-binding cassette transporter (ABCB1) inhibitors may overcome PTX resistance in breast cancer (Matsunaga et al., 2023). The AKR1C3 inhibitor tolfenamic acid (Endo et al., 2011) and siRNA treatment increase the sensitivity of non-resistant PCa cells to docetaxel (DTX), suggesting a role of AKR1C3 in DTX resistance (Shen et al., 2011).

In summary, AKR1C3’s involvement in detoxifying chemotherapeutic agents and regulating ROS levels highlights its significance in chemotherapy resistance across various cancer types. Targeting AKR1C3 may offer a promising strategy to enhance the effectiveness of chemotherapy and improve patient outcomes.

### 5.3 Endocrine therapy resistance

Endocrine therapy, a crucial method in cancer treatment, employs steroid drugs to alter the conditions necessary for tumor development, resulting in the suppression of tumor progression. Unlike chemotherapy, endocrine therapy offers precise therapeutic effects, minimal toxicity, and ease of use.

In the context of prostate carcinoma treatment, endocrine therapy includes orchiectomy and anti-androgen drugs such as bicalutamide and enzalutamide. However, resistance to potent anti-androgen drugs like enzalutamide and abiraterone acetate poses a growing challenge in advanced PCa treatment (Kafka et al., 2020). AKR1C3 has been identified as a key player in endocrine drug resistance, particularly in the context of enzalutamide resistance. Studies demonstrate that AKR1C3 is overexpressed in enzalutamide-resistant PCa cells, contributing to disease progression and resistance to enzalutamide. Targeting intracrine androgens and AKR1C3 has been proposed as a strategy to overcome enzalutamide resistance and improve the survival of advanced PCa patients (Liu et al., 2015).

Moreover, the AKR1C3/AR-V7 axis has been implicated in cross-resistance, where enzalutamide- and abiraterone-resistant PCa cells show further resistance to apalutamide and darolutamide. Knockdown of AR-V7 or targeting AKR1C3 has been suggested to resensitize resistant cells to these treatments, providing potential avenues to address drug resistance (Zhao et al., 2020).

In the context of breast cancer, which involves the use of anti-estrogen agents and aromatase inhibitors in hormone receptor-positive cases, AKR1C1, AKR1C2, and AKR1C3 have been found to be upregulated in tamoxifen-resistant breast cancer cells. This upregulation is associated with increased resistance to tamoxifen, highlighting the role of AKR1C family members in endocrine therapy resistance (Xu et al., 2021).

In conclusion, understanding the mechanisms involving AKR1C3 in endocrine therapy resistance, particularly in prostate and breast cancers, is critical for developing targeted therapies that can overcome drug resistance and improve treatment outcomes.

### 5.4 Targeted therapy resistance

Targeted therapy is a treatment strategy that employs drugs designed to precisely recognize and attack malignant tumor cells, offering a more focused therapeutic effect with fewer side effects compared to traditional treatments. These drugs include small molecule inhibitors, monoclonal antibodies, and antibody-conjugated drugs. However, resistance to targeted therapy remains a significant challenge.

In HCC, AKR1C3 has been implicated in resistance to sorafenib, a targeted therapy used in the treatment of HCC. AKR1C3 upregulation was found to enhance cell survival in response to sorafenib, while the removal of AKR1C3 restored sensitivity to sorafenib. AKR1C3 was associated with the metabolic shift from fatty acid oxidation (FAO) to glycolysis, promoting sorafenib resistance in HCC cells (Wu et al., 2022). High expression of AKR1C3 in sorafenib-resistant patients correlated with poor prognosis. The combination of AKR1C3 inhibition and sorafenib was suggested to have a more significant impact on HCC treatment, potentially overcoming resistance.

In chronic myeloid leukemia (CML), AKR1C3 has been linked to resistance to imatinib, a tyrosine kinase inhibitor used as a first-line targeted drug. High expression of AKR1C3 increased resistance to imatinib in CML cells and mouse models. Combining imatinib with indomethacin, a chemical inhibitor of AKR1C3, significantly prolonged mouse survival and reduced splenomegaly, indicating that AKR1C3 suppression has the potential to enhance imatinib treatment. Additionally, miR-379-5p, which is downregulated in the bone marrow microenvironment, was found to inhibit AKR1C3, suggesting a novel miR-379-5p/AKR1C3/ERK signaling axis in imatinib resistance in CML (Pan et al., 2021).

Understanding the role of AKR1C3 in resistance to targeted therapies is crucial for developing strategies to overcome resistance and improve the efficacy of these treatments. Targeting AKR1C3 in combination with existing targeted therapies may represent a promising approach to enhance treatment outcomes.

## 6 Emerging AKR1C3 inhibitors

**FIGURE 5:**
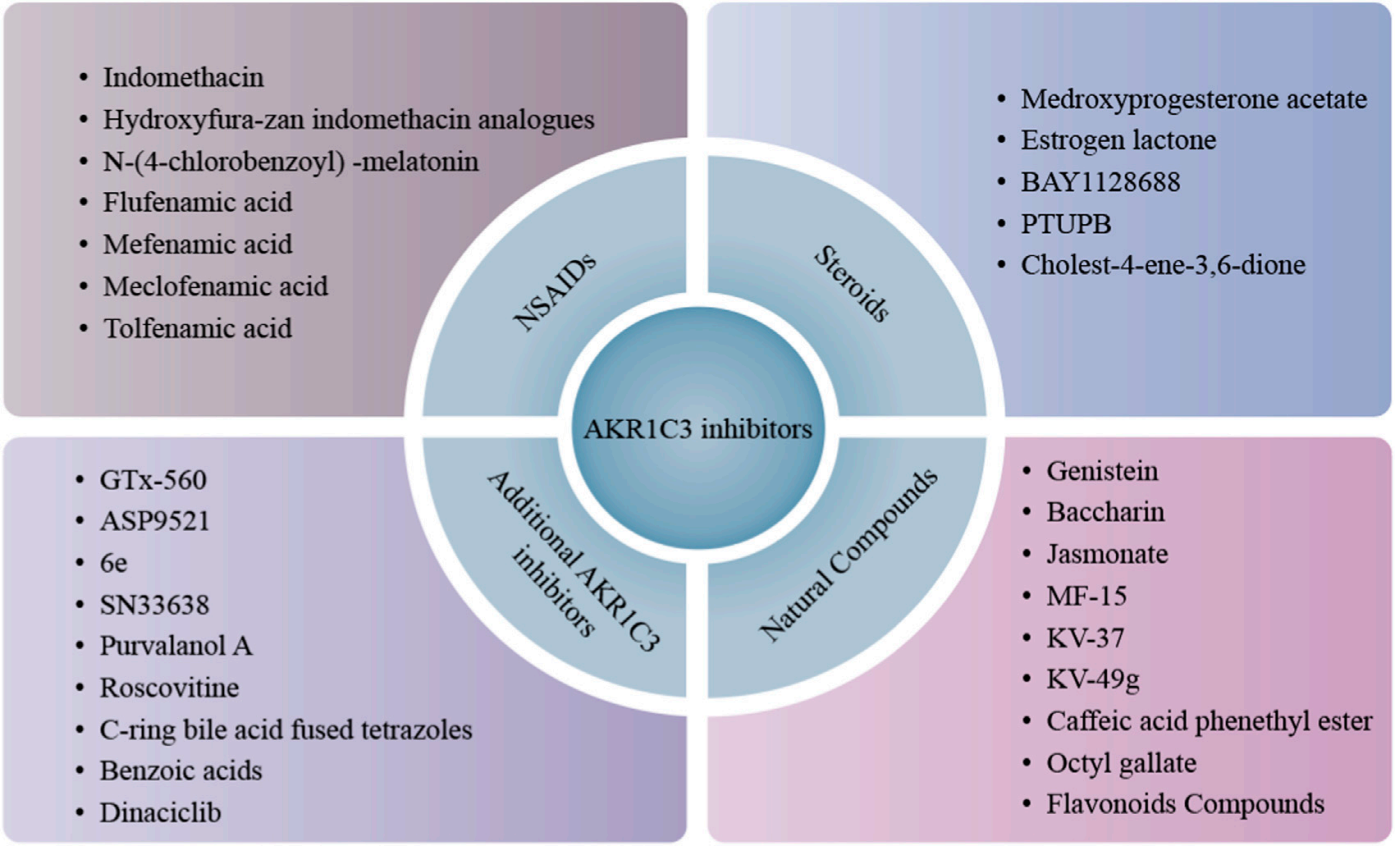
Classification of AKR1C3 inhibitors. AKR1C3 inhibitors are divided into three main categories, NSAIDs, steroids and natural compounds. Besides these, increasing small molecules are being studied and found to possess AKR1C3 inhibition potency and further expand AKR1C3 inhibitor library, like GTx-560, ASP9521, 6e, etc.

Contemporary evidence indicates the overexpression of AKR1C3 in tumors, implicating its role as an oncogene in carcinogenesis and cancer progression. Furthermore, heightened AKR1C3 expression is notably associated with an unfavorable prognosis and resistance to anti-cancer therapies. This underscores the potential significance of inhibiting AKR1C3 expression as a promising strategy to overcome drug resistance. Consequently, a burgeoning body of research has been dedicated to the exploration of AKR1C3 inhibitors with the aim of enhancing therapeutic interventions in cancer. The current landscape of AKR1C3 inhibitors has been succinctly summarized in Figure 5.

### 6.1 Non-steroidal anti-inflammatory drug

Numerous small molecules have been devised to target the enzymatic activity of AKR1C3, presenting an opportunity to disrupt its involvement in carcinoma progression and resistance to treatment.

#### 6.1.1 Indomethacin

Indomethacin, classified as a non-steroidal anti-inflammatory drug (NSAIDs) with applications in reducing fever, pain, and inflammation, serves as an inhibitor of both cyclooxygenase and AKR1C3. In patients undergoing radical prostatectomy (RP) for high-risk PCa, indomethacin demonstrates inhibition of the steroidogenic enzyme AKR1C3, particularly when used in combination with anti-androgen blockade. It exhibits selectivity over AKR1C1/AKR1C2, making it a noteworthy candidate (Graham et al., 2021).

Bioinformatic analysis of indomethacin-treated resistant cells highlights its significant activation of the unfolded protein response, p53, and apoptosis pathways. Concurrently, it suppresses cell-cycle, Myc, and AR/ARV7 pathways. The targeting of AKR1C3 with indomethacin results in a notable reduction of AR/AR-V7 protein expression both *in vitro* and *in vivo*, achieved through the activation of the ubiquitin-mediated proteasome pathway (Liu et al., 2019).

In the context of enzalutamide-resistant PCa cells, AKR1C3 inhibition by either shRNA or indomethacin demonstrates resensitization to enzalutamide. The combination of indomethacin and enzalutamide proves effective in significantly inhibiting enzalutamide-resistant PCa xenograft tumor growth, substantiating its potential in clinical applications (Liu et al., 2015). A clinical trial investigating the combination of enzalutamide and indomethacin for the treatment of recurrent or metastatic PCa is currently underway (NCT02935205).

While indomethacin exhibits therapeutic promise, its application in CRPC is limited due to side effects associated with chronic COX inhibition. Nonetheless, indomethacin serves as a crucial lead compound for the development of selective AKR1C3 inhibitors without concurrent COX activity (Liedtke et al., 2013).

#### 6.1.2 Indomethacin analogues

The initial success of indomethacin as an AKR1C3-selective inhibitor prompted further exploration into the development of compounds with enhanced selectivity and therapeutic efficacy. Indomethacin’s strong preference for inhibiting AKR1C3 over other isoforms laid the foundation for the investigation of indomethacin analogues (Byrns et al., 2008).

In a study led by Michael and colleagues, a series of NSAIDs and indomethacin analogues were examined for their inhibitory effects on AKR1C enzymes. Through structural modifications, the compound N-(4-chlorobenzoyl)-melatonin (CBM) was synthesized. CBM effectively prevented the inhibition of COX-1, COX-2, AKR1C1, and AKR1C2 while retaining AKR1C3 inhibition, making it a valuable molecular probe for studying AKR1C3’s role in breast cancer signaling and proliferation (Byrns and Penning, 2009). Indomethacin and CBM exhibited significant inhibition of the reduction of Δ4-androstene-3,17-dione by AKR1C3, with IC_50_ values of 8.5 and 11.4 μM, respectively. Importantly, they showed weak inhibition of progesterone reduction catalyzed by AKR1C1, highlighting their specificity (Byrns et al., 2008). However, CBM’s limited therapeutic potential is attributed to its poor solubility and bioavailability.

Lolli et al. introduced a novel generation of AKR1C3 inhibitors, hydroxyfurazan indomethacin analogues 1 and 2. These compounds, designed using a bioisosteric approach, replaced the carboxylic acid function of indomethacin with hydroxylated azoles. Analogues 1 and 2 exhibited superior potency (IC_50_ = 0.30 and 0.94 µM, respectively), up to 90 times more selective for AKR1C3 over AKR1C2, and demonstrated higher efficacy than indomethacin without off-target effects on AKR1C2 and COX1/2 (Lolli et al., 2019). These analogues, particularly analogue 1, effectively dose-dependently suppressed the proliferation of prostate and colorectal cancer cells with overexpressed AKR1C3.

#### 6.1.3 N-phenylanthranilic acids

Among the compounds explored as AKR1C3 inhibitors, Flufenamic acid (FLU), a sort of NSAIDs, has demonstrated potent inhibition of AKR1C3 activity (IC_50_ = 8.63 µM). However, its inhibitory effects are non-selective and lead to off-target effects on COX (Pippione et al., 2017; Pippione et al., 2018). In a study led by Zhuo and colleagues, FAA significantly downregulated the expression of both AKR1C1 and AKR1C3 in murine metastatic breast tumor cells (4T1/luc) and human HCC cells (HepG2), indicating a lack of selectivity over AKR1C1 (Li et al., 2020). Furthermore, a benzisoxazole derivative, designed through a scaffold hopping approach from FAA, displayed improved isoform selectivity as an AKR1C3 inhibitor over AKR1C1. Despite this enhancement, the inhibition potency still remained in the mid-nanomolar range (Pippione et al., 2018).

The AKR1C family’s association with drug resistance to cis-diamminedichloroplatinum (II) (CDDP) and 5-FU is noteworthy. Mefenamic acid (a 2,3-dimethyl derivative), a widely used NSAIDs and known AKR1Cs inhibitor with an IC_50_ value of 0.3 µM against AKR1C3, was easy to synthesize and inhibited AKR1C enzymes but not COX-1 or COX-2 in the pattern of competitive inhibitors of AKR1C enzymes (Penning et al., 2006; Flanagan et al., 2012). Besides, it also demonstrated the ability to increase sensitivity to CDDP and 5-FU, suggesting its potential in overcoming anticancer drug resistance (Shiiba et al., 2017), oncology reports. Additionally, the combination of simvastatin and meclofenamic acid, another AKR1C3 inhibitor (IC_50_ = 0.7 µM) (Flanagan et al., 2012), exhibited enhanced inhibition of cell proliferation and migration while inhibiting Akt activation. This combination holds promise as an effective strategy for treating castration-resistant PCa (Sekine et al., 2018). Similarly, tolfenamic acid, an AKR1C3 inhibitor, restored sensitivity to daunorubicin in human leukemic U937 cells (Matsunaga et al., 2014).

### 6.2 Steroids

Steroids, particularly medroxyprogesterone acetate (MPA) and estrogen lactone (EM1404), have demonstrated potent inhibition of AKR1C3, as indicated by low micromolar Ki values of 0.5 μM. However, their application is hindered by a lack of isoform selectivity. Notably, MPA, a contraceptive steroid, exhibits AKR1C3 inhibition at doses higher than those used for its primary indications, revealing limitations in its practical use (Khanim et al., 2014). A phase I clinical trial (NCT02434640) investigated the safety, tolerability, and pharmacokinetics of BAY1128688, a steroidal inhibitor of AKR1C3. Despite promising aspects, a phase II clinical trial (NCT03373422) focused on treating endometriosis was terminated due to hepatotoxicity resulting from nonspecific AKR1D1 inhibition (Rizner and Penning, 2020).

In a recent study, 4-(5-phenyl-3-(Short et al.)-pyrazol-1-yl)-benzenesulfonamide (PTUPB) emerged as a novel AKR1C3 inhibitor, surpassing indomethacin and celecoxib in restraining AKR1C3 activity and suppressing CRPC cell growth. PTUPB, in combination with enzalutamide, exhibited synergistic effects in tumor suppression and gene signature regulation, offering potential benefits through the blockade of AR/AR-V7 signaling. This combination showed efficacy in inhibiting the growth of castration-relapsed VCaP xenograft tumors and patient-derived xenograft organoids (Yang et al., 2023).

Cholest-4-ene-3,6-dione (KS), a steroid identified through molecular docking studies, displayed an antagonistic role against AKR1C3 (IC_50_ = 30 µM). KS exhibited similar binding orientation to indomethacin, indicating its potential as an inhibitor. Beyond inhibition, KS arrested cell cycle progression in the G1 phase, enhanced the expression of p53 and NFκB, induced caspase-12, 9, and 3 processing, and downregulated Bcl-2 expression. The findings suggest that KS serves as a molecular scaffold for developing more specific, potent, and selective small molecule inhibitors against AKR1C3 (Sali et al., 2020).

### 6.3 Natural products: exploring AKR1C3 inhibitory compounds in cancer therapy

The investigation of natural compounds with inhibitory properties against AKR1C3 is gaining prominence for its potential in cancer therapy. Noteworthy examples include:

Genistein: An active component of soy isoflavones, genistein exhibits inhibitory effects on CRPC cell proliferation, *in vivo* tumorigenesis, and CRPC progression by suppressing AKR1C3. Its synergistic effects with AKR1C3 inhibitors enhance its potential as a promising therapeutic agent (Yu et al., 2023).

Baccharin (3-prenyl-4-(dihydrocinnamoyloxy) cinnamic acid): Derived from Brazilian propolis, baccharin is a natural product with a unique structure, displaying high potency and isoform-selective competence in suppressing AKR1C3 activity (IC_50_ = 0.10 µM, 510-fold selectivity for AKR1C3 over AKR1C2) (Zang et al., 2015). Structural insights highlight specific interactions contributing to its inhibition potency and selectivity, making it a promising avenue for further development (Endo et al., 2012; Endo et al., 2014; Verma et al., 2016).

Jasmonate: Both jasmonic acid (JA) and methyl jasmonate (MeJ) function as inhibitors of AKR1C enzymes. JA exhibits potent inhibition of recombinant AKR1C proteins, while MeJ demonstrates efficient inhibition of cellular AKR1C3 activity and expression (Li et al., 2018).

MF-15: A compound derived from a plant herb, MF-15 exhibits significant inhibition potency on AKR1C3 and AR signaling, reducing the expression of both AR and AKR1C3. It demonstrates a dual effect by inhibiting androgen biosynthesis and the AR signaling cascade in tumors (Kafka et al., 2020).

(E)-3-(4-(3-methylbut-2-en-1-yl)-3-(3-phenylpropanamido) phenyl) acrylic acid (KV-37): KV-37, a cinnamic acid derivative, acts as a novel and potent AKR1C3 inhibitor with highly selective potency and stability (109-fold over AKR1C2, IC_50_ = 66 nM, t1/2 > 240 min) (Verma et al., 2016; Verma et al., 2018). Its second-generation counterpart, KV-49g, stands out as the most effective and selective AKR1C3 inhibitor known, overcoming resistance to anti-cancer agents apalutamide and darolutamide (Verma et al., 2019; Morsy and Trippier, 2020).

Phenolic Acid Derivatives: Phenolic acid derivatives, such as caffeic acid phenethyl ester (CAPE) and octyl gallate (OG), show effective inhibition of AKR1C3 among a series of analogs (IC_50_ = 3.71 µM and 2.56 µM, respectively). Understanding their molecular interactions may pave the way for the development of phenolic acid-based AKR1C3 inhibitors (Endo et al., 2012; Li et al., 2016).

Flavonoids Compounds: One kind of flavonoids compounds which were effective and selective AKR1C3 inhibitors, 2′-hydroxyflavanone, showed the most inhibition potency towards AKR1C3 (82.5% inhibition, IC_50_ = 0.3 µM). And Its selectivity for AKR1C3 is 20 times that of AKR1C2 (Skarydova et al., 2009). Identified as a specific AKR1C3 inhibitor, it restored cytotoxicity to daunorubicin and idarubicin in the lung adenocarcinoma A549 cell line with high endogenous expression of AKR1C3 (Hofman et al., 2014).

These natural products present diverse opportunities for the development of AKR1C3 inhibitors, contributing to the evolving landscape of cancer therapy.

**FIGURE 6:**
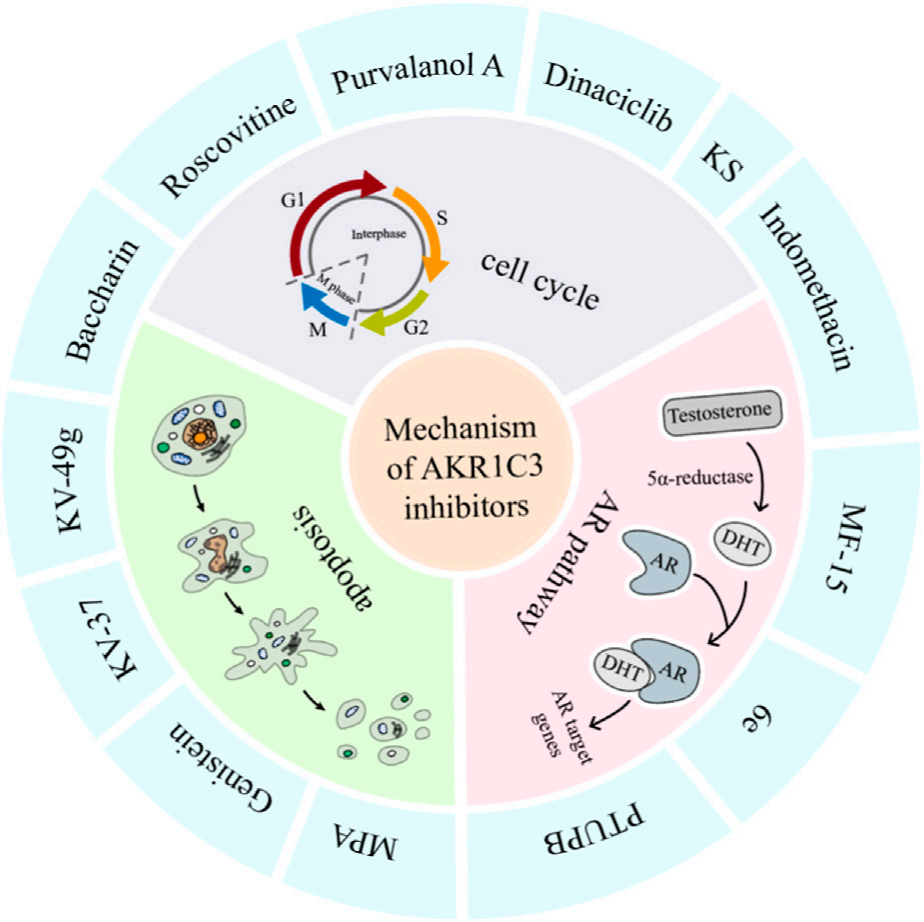
Anti-tumor mechanisms of AKR1C3 inhibitors. AKR1C3 inhibitors exerts their potency against tumors via multifarious mechanism, among which prevalingly were cell cycle, AR pathway, and apoptosis. Of the above inhibitors, indomethacin impacts both cell cycle and AR pathway, and baccharin affects both cell cycle and apoptosis.

In conclusion, a diverse array of small molecules has been devised to specifically target the enzymatic activity of AKR1C3, offering a promising avenue for impeding its role in carcinoma progression and treatment resistance. This review offers a thorough and encompassing overview of AKR1C3 inhibitors, spanning both preclinical investigations and clinical trial stages. For detailed insights, please consult Table 1. Additionally, potential mechanisms of action for AKR1C3 inhibitors have been elucidated in Figure 6.

**TABLE 1:** Comprehensive overview of AKR1C3 inhibitors at preclinical stages.

Inhibitor name	Type	Specificity	Stage	IC_50_ value	Characteristic	References
CBM	NSAIDs	AKR1C3	Preclinical	11.4 μM	without COX inhibition, poor solubility and bioavailability	Byrns et al. (2008), Byrns and Penning (2009)
Hydroxyfurazan indomethacin analogues	NSAIDs	AKR1C3	Preclinical	0.30 μM	without off-target effects on AKR1C2 and COX1/2	Lolli et al. (2019)
FLU	NSAIDs	pan-AKR1C	Preclinical	8.63 μM	non-selective inhibitory effects, COX off-target effects	Penning (2017), Pippione et al. (2017), Pippione et al. (2018)
Mefenamic acid	NSAIDs	AKR1Cs	Preclinical	0.3 μM	easy to synthesize, similar bind with FLU, without COX inhibition	Penning et al. (2006), Flanagan et al. (2012)
Meclofenamic acid	NSAIDs	AKR1Cs	Preclinical	0.7 μM	similar bind with FLU	Flanagan et al. (2012)
MPA	steroids	pan-AKR1C	Preclinical	8.4 μM	lack of isoform selectivity, higher doses	Khanim et al. (2014)
PTUPB	steroids	AKR1C3	Preclinical	0.49 nM	dual COX-2/sEH inhibitor, superior AKR1C3 binding	Yang et al. (2023)
KS	steroids	AKR1C3	Preclinical	30 µM	similar binding orientation to indomethacin	Sali et al. (2020)
Genistein	natural products	AKR1C3	Preclinical	6.3 μM	low bioavailability	Yu et al. (2023)
Baccharin	natural products	AKR1C3	Preclinical	0.10 µM	high potency and isoform-selective competence	Endo et al. (2012), Endo et al. (2014), Zang et al. (2015), Verma et al. (2016), Penning (2017)
Methyl jasmonate	natural products	pan-AKR1C	Preclinical	33.42 µM	high therapeutic doses	Davies et al. (2009), Li et al. (2018)
MF-15	natural products	AKR1C3	Preclinical		dual inhibition on AKR1C3 and AR signaling	Kafka et al. (2020)
KV-37	natural products	AKR1C3	Preclinical	66 nM	potent, isoform-selective and hydrolytically stable	Verma et al. (2016), Verma et al. (2018)
CAPE	natural products	AKR1Cs		3.71 µM	strong inhibition potency without selectivity	Li et al. (2016)
OG	natural products	AKR1Cs		2.56 µM	strong inhibition potency without selectivity	Li et al. (2016)
2′-hydroxyflavanone	natural products	AKR1C3	Preclinical	0.3 µM	high potency and selectivity of AKR1C3	Skarydova et al. (2009)
GTx-560		AKR1C3	Preclinical		dual AKR1C3 and AR inhibitors	Yepuru et al. (2013), Wangtrakuldee et al. (2019)
6e	mansonone derivative	AKR1C3	Preclinical	1.94 μM	selectivity, AKR1C3-dependent	Zhou et al. (2020)
SN33638	non-carboxylate	AKR1C3	Preclinical		partial inhibition	Heinrich et al. (2013), Yin et al. (2014)
Purvalanol A	CDK inhibitor	AKR1C3	Preclinical	6.58 μM	non-competitive inhibitors, inhibit human recombinant AKR1C3	Hikita et al. (2010), Novotna et al. (2018b)
Roscovitine	CDK inhibitor	AKR1C3	Preclinical	2.15 μM	non-competitive inhibitors, inhibit human recombinant AKR1C3	Marozin et al. (2010), Novotna et al. (2018b)
C-Ring Bile Acid Fused Tetrazoles	steroidal bile acid fused tetrazoles	AKR1C3		7 μM	selectivity for AKR1C3 over KR1C2, no off-target affinity for ERα or AR receptors	Marinovic et al. (2023)
3-(3,4-Dihydroisoquinolin-2(1H)-ylsulfonyl) Benzoic Acids		AKR1C3		6.1 nM	greater inhibitory potency in AKR1C3 over AKR1C2	Kong et al. (2022)
3 ((4-Nitronaphthalen-1-yl) Amino) benzoic Acid 1	naphthyl derivative	AKR1C3	Preclinical	0.086 µM	AKR1C3 competitive inhibitor and AR antagonist	Wangtrakuldee et al. (2019)
Dinaciclib	CDK inhibitor	AKR1C3	Preclinical	0.23 µM	a tight-binding inhibitor of AKR1C3	Novotna et al. (2018a), Novotna et al. (2018b)
Olaparib	Poly (ADP-ribose) polymerase	AKR1C3	Preclinical	2.48 µM	high inhibitory affinity toward AKR1C3	Tavares et al. (2020)
S07-2010		pan-AKR1C	Preclinical	0.19 µM	sub-micromolar potency toward all the AKR1Cs in the study	He et al. (2022)

## 7 Combination therapies of AKR1C3 inhibitors and antineoplastic therapies

AKR1C3, recognized not only as a biomarker for cancer progression and prognosis but also as a novel target for overcoming therapeutic resistance, presents opportunities for combination therapies with various existing treatments. This includes endocrine agents, chemotherapy, and immunotherapy, suggesting potential synergistic effects. The identification of patient subsets most likely to benefit from AKR1C3 inhibition is imperative for tailoring personalized treatment strategies.

### 7.1 Bezafibrate and medroxyprogesterone acetate combination

Bezafibrate, known for promoting PGD2 generation by increasing ROS and activating the lipid peroxidation pathway, has demonstrated promising results. In collaboration with MPA, this combination led to a significant accumulation of PGD2 and 15d-PGJ2. This accumulation correlated with growth arrest, apoptosis, and cell differentiation in AML cell lines and primary AML cells, emphasizing the potential of combining AKR1C3 inhibitors with established therapies for enhanced outcomes (Murray et al., 2010).

### 7.2 Pan-AKR1C inhibitor S07-2010 and chemotherapy

The pan-AKR1C inhibitor S07-2010 exhibited notable inhibitiory effect (IC50 = 0.19 μM) and adjuvant potency, surpassing other compounds studied in the context of MCF-7/DOX and A549/DDP cells. Combining 25 μM DOX with 10 μM S07-2010 resulted in a 29% reduction in cell viability. Additionally, the combination demonstrated increased cell apoptosis and decreased proliferation when S07-2010 was used in conjunction with cisplatin (DDP), illustrating the potential of AKR1C3 inhibitors to enhance the efficacy of chemotherapy (He et al., 2022).

### 7.3 Olaparib and anthracyclines reversal

Olaparib, identified by Tavares et al., exhibited significant inhibition potency against AKR1C3 (IC_50_ = 2.48 µM) at the cellular level in HCT116 cells. The inhibition of AKR1C3 by olaparib demonstrated a reversal of resistance to anthracyclines, suggesting a potential role for AKR1C3 inhibitors in overcoming resistance to certain antineoplastic agents (Tavares et al., 2020).

### 7.4 KV-37 and enzalutamide combination

KV-37, whose biological activity was AKR1C3-dependent, decreased PCa cell viability in a dose and time-dependent manner and restored cellular sensitivity to enzalutamide. Combination of KV-37 and enzalutamide retarded enzalutamide-resistant PCa cells growth and induced apoptosis that may be the major mechanism of combination therapy against tumor (Verma et al., 2018).

These findings underscore the potential of combining AKR1C3 inhibitors with established antineoplastic therapies, offering a multifaceted approach to enhance treatment efficacy and address therapeutic resistance in various cancer contexts.

## 8 Clinical trails of AKR1C3 inhibitors and prodrugs

AKR1C3 participates in various biological processes and signalling pathways. And it is known to be upregulated in certain types of tumors and is associated with tumor progression and drug resistance to further confirm its potential as a therapeutic target. However, there is relatively limited clinical trials are investigating the efficacy of AKR1C3 inhibitors and prodrugs as anti-cancer treatment strategies. Meanwhile it should be noted that research on AKR1C3 in clinical trials is still in its early stages, and further investigation is needed to assess its safety and effectiveness. More detailed information about clinical trials of AKR1C3 were summarized in Table 2.

**TABLE 2:** Clinical trial related to AKR1C3 inhibitors and prodrugs.

ClinicalTrials.gov ID	Study phase	Drug	Classification/Function/Role	Disease	Objective	Status
NCT03592264	Clinical phase Ⅰ/Ⅱ	OBI-3424	AKR1C3 prodrug	Pancreatic adenocarcinoma	evaluate the safety, tolerability, MTD/RP2D, PK, and preliminary efficacy of OBI-3424	Recruiting
NCT04315324	Clinical phase Ⅱ	OBI-3424	AKR1C3 prodrug	T-ALL	assess the response rate of OBI-3424 (CR or CR with CRi)	Active, not recruiting
NCT06239155	Clinical phase Ⅰ/Ⅱ	AST-3424	AKR1C3 prodrug	HCC	evaluate the safety, tolerability, PK, and preliminary efficacy of AST-3424	Recruiting
NCT02935205	Clinical phase Ⅰ/Ⅱ	Indomethacin	AKR1C3 inhibitor	Prostate carcinoma	assess the toxicity of indomethacin and enzalutamide	Recruiting
NCT02434640	Clinical phase Ⅰ	BAY1128688	AKR1C3 inhibitor	Endometriosis	investigate the safety, tolerability and PK of BAY1128688	Completed
NCT03373422	Clinical phase Ⅱ	BAY1128688	AKR1C3 inhibitor	Endometriosis	test effect of BAY1128688 in relieving pain and optimal dose	Terminated (due to hepatotoxicity)
NCT01352208	Clinical phase Ⅰ/Ⅱ	ASP9521	AKR1C3 inhibitor	CRPC	investigate the safety, tolerability and initial anti-tumor activity of ASP9521	Terminated (owing to the results of a P1 study)
NCT00862134	Clinical phase Ⅱ	PR104	AKR1C3 prodrug	NSCLC	estimate RR of PR104/docetaxel	Terminated
NCT01037556	Clinical phase Ⅰ/Ⅱ	PR104	AKR1C3 prodrug	Acute Leukemia	determine the toxicities and recommended dose of PR104 to relapsed/refractory AML and ALL patient	Unknown
NCT00862082	Clinical phase Ⅰ/Ⅱ	PR104	AKR1C3 prodrug	HCC	MTD of PR104 in combination with standard dose Sorafenib	Terminated (due to poor tolerance in HCC patients)
NCT02347813	Clinical phase Ⅱ	Pioglitazone	AKR1C3 prodrug	Skin squamous cell carcinoma	detect the statistically change in SCC tumor numbers	Completed
NCT04954599	Clinical phase Ⅰ/Ⅱ	CP-506 (HAP)	Hypoxia activated prodrug	Unspecified Solid Tumor	study the safety and pharmacokinetics of intravenous infusion of CP-506	Recruiting

ALL: acute lymphocytic leukemia; AML: acute myelogenous leukemia; CR: complete remission; CRi: incomplete count recovery; MTD: maximum tolerated dose; PCOS: polycystic ovary syndrome; PK: pharmacokinetics; RR: response rate; SCC: squamous cell carcinoma; SNP: single nucleotide polymorphism.

## 9 Concluding remarks and perspectives

AKR1C3, a versatile enzyme widely distributed in liver, prostate, and breast tissues, assumes a critical role in steroid hormone and prostaglandin metabolism. Elevated expression levels, particularly observed in prostate and liver cancers, designate AKR1C3 as a potential therapeutic target for addressing CRPC and overcoming drug resistance in HCC treatment. Nonetheless, there are several pivotal questions necessitating systematic research across diverse tumor models. Firstly, the suitability of specific AKR1C3 variants for treating hormone-dependent or hormone-independent cancers requires clarification. Secondly, the precise mechanisms for overcoming drug resistance in radiotherapy and chemotherapy demand elucidation. Thirdly, identifying and addressing bottlenecks in the clinical development of AKR1C3 inhibitors is crucial. Resolving these questions mandates comprehensive research in basic laboratories to uncover novel anticancer mechanisms of AKR1C3. For instance, AKR1C3, beyond its enzymatic function, functions as a regulator of ferroptosis-related genes (FRGs) in PCa (Liu et al., 2021). The impact of AKR1C3 on ferroptosis, an iron-dependent form of programmed cell death, involves its downregulation of ferroptosis indicators with its overexpression (Wang et al., 2023). Therefore, a thorough exploration of the detailed mechanisms of AKR1C3 in tumor biology through diverse experimental approaches is imperative. Additionally, well-designed clinical trials in suitable cancer patients are essential for expediting the development of AKR1C3 inhibitors as both single-targeted and combination therapy with current anticancer drugs. Encouragingly, ongoing advancements in anti-tumor drugs and AKR1C3 inhibitors, such as ASP9521 and BAY1128688, are progressing in clinical trials for CRPC treatment (Rizner and Penning, 2020). Nanodrug-delivery systems (NDDS), exemplified by CS-4D5/6e, exhibit enhanced inhibitory potency, leveraging improved pharmacokinetics, tumor-targeting capabilities, and responsive drug release in the tumor microenvironment (Zhou et al., 2020). Despite the promising clinical applications of NDDS, challenges remain, including optimizing drug release and addressing potential drawbacks, such as increased drug accumulation.

The multifaceted roles of AKR1C3 in carcinoma progression and therapeutic resistance underscore its significance as a therapeutic target. In a parallel study utilizing the HL-60 cell line, evidence emerged suggesting a link between the proliferative effect of AKR1C3 and the retinoic acid signaling pathway. Specifically, 9-cis-retinol demonstrated an anti-proliferative effect, likely attributed to its conversion into 9-cis-RA (Ruiz et al., 2011). This finding adds to the complexity of AKR1C3’s regulatory roles, reinforcing the need for comprehensive investigations into its molecular mechanisms and potential therapeutic implications.

The ongoing development of AKR1C3 inhibitors holds promise for addressing treatment challenges in carcinomas. Beyond the previously discussed inhibitors, various agents demonstrate inhibition potency towards AKR1C3 and expand therapeutic avenues of both hormone-dependent cancers and hormone-independent cancers such as: GTx-560, ASP9521, SN33638, etc.

Continued research efforts aim at unraveling the intricacies of AKR1C3’s contributions and refining inhibitor design are poised to reshape the landscape of carcinoma therapy, offering new avenues for improved patient outcomes.
